# Exploring horizons: expanding nursing education with immersive 360º videos

**DOI:** 10.1590/0034-7167.2025780101

**Published:** 2025-01-13

**Authors:** Vitória Talya dos Santos Sousa, Leandra Velyne Cardozo Martins, Patrícia Freire de Vasconcelos

**Affiliations:** I Universidade da Integração Internacional da Lusofonia Afro-Brasileira. Redenção, Ceará, Brazil

In the current healthcare landscape, we face challenges regarding patient safety. In the field of nursing, in particular, error rates committed by students range from 18.8% to 32.1%^(1)^. This demands the pressing need for new teaching strategies capable of reducing error rates and enabling safer, higher quality environments.

Among the available options, simulated practices stand out in training and skills improvement. Simulated scenarios are widely used throughout the world for both training and assessment of students and professionals in specific skills or more complex situations that require varied practices. However, despite being promising in amplifying these benefits, their integration with videos is little explored.

Notably, videos, in different formats and with different objectives, are widely used in nursing. However, they are often designed to convey information indirectly, placing the person watching them as a mere spectator. This results in distancing and a feeling of verticalized learning, in which these subjects would be merely receivers.

In the meantime, 360º videos are seen as a promising trend, allowing a complete view of the filmed environment and providing immersion to those watching. This technology is proving to be important for nursing education, considering that the existence of contexts, such as specific emergencies or critical care, may be inaccessible. Thus, immersion through videos is an interesting and viable alternative to contribute to theoretical-practical classes.

Although incipient, the effectiveness of learning in the field of mental health nursing has already been demonstrated, contributing to the development of students’ skills, attitudes and knowledge^(2)^. Another report in the literature, which used 360º videos in scenarios with patients with schizophrenia, demonstrated that these were useful and interesting, as they motivated and engaged students in view of the realism employed^(3)^.

Given the above, increased attention, user satisfaction, skill enhancement, and confidence in usability are beneficial in its use. However, there are still limitations of studies resulting from small sample sizes, uncontrolled testing, and lack of adequate assessment of learning outcomes. This demonstrates the need for more comprehensive research to fill these gaps.

Furthermore, it is important to recognize the challenges associated with producing and using these videos. The need for specific cameras for recording, especially when importing such equipment, requires financial resources. For a more immersive experience, other equipment may be required, such as virtual/augmented reality glasses and/or headsets, which also makes the process more expensive.

Despite the financial costs, using 360º videos with virtual reality glasses provides a more complete and immersive experience. A recent study indicated that the use of these devices promoted higher levels of a sense of presence and positive emotions compared to presentations viewed on a desktop^(4)^. However, it is important that researchers/professors assess the best way to use it and understand what best suits their reality, considering needs and available resources.

Thus, we concluded that using 360º videos provides promising and innovative benefits based on growing scientific evidence. Although more studies are needed to assess their effectiveness in comparison with other strategies, offering them to students has been shown to be positive and can contribute to more active and immersive teaching.
