# Supplementary material for: Exploring horizons: expanding nursing education with immersive 360º videos
Source: Rev Bras Enferm. 2025 Jan 13;78(1):e780101. doi: 10.1590/0034-7167.2025780101 (PMC11731845; doi:10.1590/0034-7167.2025780101)
Supplement: Translation

# Translation: Explorando horizontes: ampliando o ensino em enfermagem com vídeos imersivos em 360º

**DOI:** 10.1590/0034-7167.2025780101pt

**Authors:** Vitória Talya dos Santos Sousa, Leandra Velyne Cardozo Martins, Patrícia Freire de Vasconcelos

**Affiliations:** I Universidade da Integração Internacional da Lusofonia Afro-Brasileira. Redenção, Ceará, Brasil

No cenário atual da saúde, enfrentamos desafios em relação à segurança do paciente. Na área da enfermagem, em particular, as taxas de erros cometidos por estudantes variam entre 18,8% e 32,1%^(1)^. Isso demanda a necessidade premente de novas estratégias de ensino capazes de reduzir taxas de erro e permitir ambientes mais seguros e com maior qualidade.

Entre as opções disponíveis, as práticas simuladas destacam-se nos treinamentos e aperfeiçoamentos de habilidades. Os cenários simulados são amplamente utilizados em todo o mundo, tanto para o treinamento quanto para avaliação de discentes e profissionais em habilidades específicas ou situações mais complexas que necessitam de práticas variadas. Todavia, apesar de ser promissora em amplificar esses benefícios, sua integração com vídeos é pouca explorada.

Notavelmente, os vídeos, em diferentes formatos e com objetivos variados, são amplamente utilizados na enfermagem. Contudo, frequentemente são planejados com o intuito de transmitir informações de forma indireta, colocando a pessoa que o assiste como mero espectador. Isso resulta em distanciamento e sensação de aprendizagem verticalizada, em que esses sujeitos seriam apenas receptores.

Nesse ínterim, os vídeos em 360º são apontados como uma tendência promissora, permitindo uma visualização completa do ambiente filmado e proporcionando imersão a quem assiste. Essa tecnologia se revela como importante para o ensino da enfermagem, considerando que a existência de contextos, como emergências específicas ou cuidados críticos, pode ser inacessível. Dessa forma, a imersão por meio de vídeos é uma alternativa interessante e viável para contribuir com as aulas teórico-práticas.

Ainda que incipientes, já é demonstrada a eficácia para o aprendizado na área da enfermagem em saúde mental, contribuindo para o desenvolvimento de habilidades, atitudes e conhecimentos dos alunos^(2)^. Outro relato na literatura, que utilizou vídeos em 360º em cenários com pacientes com esquizofrenia, demonstrou que esses apresentaram-se úteis e interessantes, por motivar e engajar os discentes diante do realismo empregado^(3)^.

Diante do exposto, o aumento da atenção, satisfação do usuário, aprimoramento de habilidades e confiança na usabilidade é benéfico em sua utilização. No entanto, ainda há limitações de estudos decorrentes de amostras pequenas, testes não controlados e falta de avaliação adequada dos resultados de aprendizagem. Isso demonstra a necessidade de pesquisas mais abrangentes para preencher essas lacunas.

Além disso, é importante reconhecer os desafios associados à produção e utilização desses vídeos. A necessidade de câmeras específicas para a gravação, especialmente na importação desses equipamentos, necessita de recursos financeiros. Para uma experiência mais imersiva, poderão ser necessários outros equipamentos, como óculos de realidade virtual/aumentada e/ou *headsets*, o que também torna o processo mais oneroso.

Apesar dos custos financeiros, o uso dos vídeos em 360º com óculos de realidade virtual propicia uma experiência mais completa e imersiva. Estudo recente indicou que a utilização desses equipamentos promoveu níveis mais elevados de sensação de presença e emoções positivas, em comparação à apresentação com visualização em *desktop* ^(4)^. Entretanto, é importante que pesquisadores/professores avaliem a melhor forma de utilização e compreendam o que melhor se adequa à sua realidade, considerando necessidades e recursos disponíveis.

Dessa forma, concluímos que a utilização de vídeos em 360º proporciona benefícios promissores e inovadores por evidências científicas crescentes. Ainda que mais estudos sejam necessários para avaliar sua efetividade, em comparação com outras estratégias, ofertá-los a discentes mostra-se positivo, e pode contribuir para um ensino mais ativo e imersivo.

# Translation: Explorando horizontes: ampliando la educación en enfermería con vídeos inmersivos de 360º

**DOI:** 10.1590/0034-7167.2025780101es

**Authors:** Vitória Talya dos Santos Sousa, Leandra Velyne Cardozo Martins, Patrícia Freire de Vasconcelos

**Affiliations:** I Universidade da Integração Internacional da Lusofonia Afro-Brasileira. Redenção, Ceará, Brasil

En el escenario sanitario actual, nos enfrentamos a retos en materia de seguridad del paciente. En el área de enfermería, en particular, los índices de error cometidos por los estudiantes varían entre el 18,8% y el 32,1%^(1)^. Esto requiere una necesidad urgente de nuevas estrategias de enseñanza capaces de reducir las tasas de error y permitir entornos más seguros y de mayor calidad.

Entre las opciones disponibles destacan las prácticas simuladas en términos de formación y mejora de habilidades. Los escenarios simulados son ampliamente utilizados en todo el mundo tanto para la formación como para la evaluación de estudiantes y profesionales en habilidades específicas o situaciones más complejas que requieren prácticas variadas. Sin embargo, a pesar de ser prometedor a la hora de amplificar estos beneficios, su integración con vídeos está poco explorada.

Cabe destacar que los vídeos, en diferentes formatos y con distintos objetivos, se utilizan ampliamente en enfermería. Sin embargo, muchas veces se planifican con el objetivo de transmitir información de forma indirecta, situando a quien observa como un mero espectador. Esto resulta en un distanciamiento y una sensación de aprendizaje verticalizado, en el que estos sujetos serían sólo destinatarios.

Mientras tanto, los vídeos en 360º se consideran una tendencia prometedora, ya que permiten una visualización completa del entorno filmado y proporcionan inmersión a quienes los miran. Esta tecnología resulta importante para la educación de enfermería, considerando que la existencia de contextos, como emergencias específicas o cuidados críticos, puede ser inaccesible. Por tanto, la inmersión a través de vídeos es una alternativa interesante y viable para contribuir a las clases teórico-prácticas.

Aunque incipiente, la efectividad del aprendizaje en el área de enfermería en salud mental ya ha sido demostrada, contribuyendo al desarrollo de habilidades, actitudes y conocimientos de los estudiantes^(2)^. Otro reporte en la literatura, que utilizó videos de 360º en escenarios con pacientes con esquizofrenia, demostró que estos eran útiles e interesantes, ya que motivaron e involucraron a los estudiantes frente al realismo utilizado^(3)^.

Teniendo en cuenta lo anterior, una mayor atención, la satisfacción del usuario, la mejora de las habilidades y la confianza en la usabilidad resultan beneficiosos en su uso. Sin embargo, todavía existen limitaciones en los estudios derivadas de muestras pequeñas, pruebas no controladas y falta de una evaluación adecuada de los resultados del aprendizaje. Esto demuestra la necesidad de una investigación más exhaustiva para llenar estos vacíos.

Además, es importante reconocer los desafíos asociados con la producción y el uso de estos videos. La necesidad de cámaras específicas para grabar, especialmente cuando se importan estos equipos, requiere recursos económicos. Para una experiencia más inmersiva, pueden ser necesarios otros equipos, como gafas y/o cascos de realidad virtual/aumentada, lo que también encarece el proceso.

A pesar de los costes económicos, utilizar vídeos 360º con gafas de realidad virtual proporciona una experiencia más completa e inmersiva. Un estudio reciente indicó que el uso de este equipo promovió niveles más altos de sensación de presencia y emociones positivas en comparación con la presentación con visualización en *desktop* ^(4)^. Sin embargo, es importante que los investigadores/docentes evalúen la mejor manera de utilizarlo y comprendan qué se adapta mejor a su realidad, considerando las necesidades y los recursos disponibles.

Por tanto, concluimos que el uso de vídeos de 360º proporciona beneficios prometedores e innovadores basados en una creciente evidencia científica. Aunque se necesitan más estudios para evaluar su efectividad, en comparación con otras estrategias, ofrecerlas a los estudiantes es positivo y puede contribuir a una enseñanza más activa e inmersiva.
